# Traditional uses and practices of edible cultivated *Allium* species (fam. Amaryllidaceae) in Sweden

**DOI:** 10.1186/s13002-022-00513-z

**Published:** 2022-03-10

**Authors:** Erik de Vahl, Ingvar Svanberg

**Affiliations:** 1 Pom, Swedish University of Agricultural Sciences, Box 190, 234 22 Alnarp, Sweden; 2 Institute for Russian and Eurasian Studies, Uppsala University, Box 514, 751 20 Uppsala, Sweden

**Keywords:** Cultivated diversity, Cultural ecosystem services, Ethnobiology, Garden crops, Horticulture, Peasant gardens, Medicinal plants, Vegetables

## Abstract

**Background:**

While the utilitarian crops grown in vicarage gardens in pre-industrial Sweden have been fairly well documented, our knowledge of plants cultivated for food among the peasants and crofters is limited. Nevertheless, garden vegetables and herbs played a much more important role in the diet of the rural population from a nutritional point of view than, say, wild plants, at least in the southern part of the country. This study aims to explore the importance of edible cultivated onions, *Allium*, and their various cultivars and old landraces that were once—and in some cases still are—grown in home gardens.

**Methods:**

This study is based on documentation collected from national surveys carried out by the Swedish National Programme for Diversity of Cultivated Plants (POM), and from an intense search for references to the cultivation and use of carious onions in the historic garden literature, herbals and ethnographic records found in responses to folklife questionnaires.

**Results:**

The rural population in pre-industrial Sweden cultivated various kinds of bulb onions. They are known under various folk names, although their taxonomic affiliation has been unclear. Many folk taxa have been classified and named by their use, while other names refer to the practices associated with the cultivation system. These onions were often described as especially well suited for storage over winter. Onions have had a wide range of uses in Sweden. In some parts of Sweden, onions were eaten during church service in order to keep the churchgoers awake. Several types of onion have commonly been used as condiments in pickled herring dishes, spreads, sauces, foods made of blood and offal, dumplings, meat dishes and soups. Garlic was used for medicinal and magical purposes, as well as for ethnoveterinary medicine. Onion skins have traditionally been used for dyeing eggs at Easter.

**Conclusion:**

Genetic diversity of vegetables and garden crops represents a critical resource to achieve and maintain global food security. Therefore, ethnobiologists studying agricultural societies should place more focus on old landraces, cultivars and cultivation practices in order to understand the importance of garden crops for a society. They are an important element of sustainability.

## Introduction

Although some wild species of *Allium* have historically been harvested as spring vegetables and chicken feed in southern Sweden [1, 2], most onions consumed in Sweden are bulb onions, which have a long history as cultivated plants. We do not know for certain when the cultivated species reached northern Europe. Peasants’ home gardens are known to have existed on the Scandinavian Peninsula since the Viking Age [3, 4]. Special fenced gardens for *Allium* cultivation, known as *laukagarð*’ (‘onion gardens’), are mentioned in the Old Norse laws, sagas and chronicles [5]. Historical records confirm that plants of the genus *Allium* have been part of the everyday diet in Sweden since at least the fourteenth century, when the cultivation of onions was regulated in the Country Law of Magnus Eriksson from around 1341 [6]. While growing sites for onions in Iceland and Norway in the sources were distinguished from vegetable gardens for crops categorised as ‘kale’, the Swedish Medieval provincial laws include onions as a specific category of crops that were illegal to take from another person’s kale or herb garden [6].

The absence of onion seeds in archaeological remains has been interpreted to mean that onions were mainly vegetatively propagated in the Nordic region [7]. This has been challenged by the discovery of onion seed remains from trade in the Baltic Sea [8]. The use of onion seed in the royal gardens in Stockholm is recorded from the sixteenth century [9]. The import of onions from Danzig (contemporary Gdańsk) is described from the same period [10]. Swedish records of custom cleared goods from the sixteenth and seventeenth centuries show that onions labelled as Dutch (*Holländsk*) were imported and sometimes distinguished from onions from Stralsund [11]. Beside Dutch onions, Portuguese and Spanish onions are also mentioned in the sources, such as in a list of plants grown in a university garden in Uppsala from 1638 [12]. Portuguese onion is mentioned in customs documents from Stockholm as early as 1534 [13].

There is little information on the diversity of species grown in Swedish rural home gardens before the nineteenth century. However, recent research has shown that these gardens were numerous [14] and diverse [15]. A certain kind of onion, sometimes called *Johannisslök*, ‘Saint John’s onion’, is often described in older garden literature and has for a long time had an unclear taxonomic affiliation. In the travel diary of the Swedish naturalist Carl Linnaeus from province of Dalecarlia in 1734, *Johannisslök* is mentioned as a plant brought to church as a snack [16]. In later records, Linnaeus and his contemporaries used this vernacular name for both *Allium fistulosum* L. and *Allium schoenoprasum* var. *sibiricum* (L.) Hartm. It has also been a synonym for shallot and potato onion, *Allium cepa* L. Aggregatum-Group [17].

In nation-wide surveys conducted by the Swedish National Programme for Diversity of Cultivated Plants (POM) in 2002–2010, it was discovered that heirloom varieties of vegetatively propagated onions called *potatislök* (‘potato onions’), *scharlottenlök* (‘shallots’) or by various geographical/local names were preserved within households or families as heritage plants. This was noted from all over the country [18]. These heirloom onions were often described as especially well suited for storage over winter. They were in fact genetically diverse and believed to originate from plant material that had been cultivated in the countryside for a long time [19].

The plant material collected and preserved in the Swedish National Gene Bank for Vegetatively Propagated Horticultural Crops as *A. cepa* L. Aggregatum-Group cannot be distinguished from cultivars known as shallots. Genetic examination shows that similar genotypes can be found in many European countries [20]. The relatively recent inclusion of shallots in the species *A. cepa* L., and the fact that the origin of the shallot is unknown, has contributed to an inconsistent use of names across different regions and countries [21–23]. According to popular belief, the shallot was thought to originate from the ancient Canaanite city of Ashkelon (hence its former scientific name *Allium ascalonicum* L., in use until 2010). This opinion was based on Linnaeus’ belief that his pupil Frederic Hasselquist had found the species growing wild in the area [24], but the specimen collected was later found to be another species [23].

A review of the literature reveals that cultivars of shallots were imported, cultivated and distributed in Sweden in the 1830s and 1840s [17, 22]. The landrace cultivar 'Leksand', also known locally as *Leksandslök* (‘onion from Leksand’), was grown in the garden of the Swedish Garden Association in 1859, and several cultivars were known at the time in France, Germany and England.

An early introduction to Sweden called *Egyptisk potatislök*, ‘Egyptian potato onion’, in the nineteenth century was often confused with the tree onion, *Allium* × *proliferum* [22]. The tree onion was also for a long time erroneously believed to be of Egyptian origin [25].

Until the mid-nineteenth century, autumn planting was often proposed in the instructions given in Swedish garden literature [18]. However, according to the surveys carried out by POM, it was obvious that neither these descriptions nor the recorded plant names fully aligned with the practices and traditions as described by the donors of plant material in POM’s inventory.

### The aim of the study

While economic crofts and plants grown in vicarage gardens in pre-industrial Sweden are fairly well documented, our knowledge about plants cultivated for food among the peasants and crofters is limited [15, 26, 27]. Our objective is to deepen knowledge of uses and practices associated with the most common species of *Allium* grown by the Swedish pre-industrial rural population. Efforts on the conservation of genetic resources, undertaken as part of the FAO International Convention on Plant Genetic Resources for Food and Agriculture (adopted in 2001), require increased knowledge not only of the biological values of cultivated plants, but also of other values linked to cultural history. These values are variously referred to as ‘bio-cultural values’ [28], ‘cultural ecosystem services’ and ‘social values’ [29].

In this article, we aim to deepen knowledge of uses and practices associated with the most common species of *Allium* grown by the pre-industrial Swedish peasantry, and how growing practices for edible *Alliums* as documented in historical literature correspond to records from early twentieth-century folklife questionnaires and modern inventories. The questionnaires as source material for garden history may provide a link between historical written sources and documentation from a national inventory compiled in the twenty-first century, earlier described in the Swedish publications *Klint Karins kålrot och Mor Kristins böna* [30], and *Kulturarvsväxter för framtidens mångfald* [18]. The archival material contains knowledge connected to everyday life and practices in Swedish rural society in the first half of the twentieth century before large-scale urbanisation took place, as perceived and remembered by scientists and respondents. By comparing knowledge of a particular crop obtained from different sources, we hope not only to bring together preserved historical plant material with relevant historical knowledge, but also to discuss how a broader approach to garden history might be developed in order to better understand practices seldom described in the literature.

In order to interpret old records, it is necessary to know that the word *kål* (‘kale’) in historical sources often includes all kinds of potherbs and kitchen vegetables grown for their leaves, stalks and buds [31]. We have earlier described how different species of *Alliums* were used in similar ways and sometimes classified by their use, rather than by their taxonomy [17]. These categories are referred to as ‘popular plant families’ [31].

We aim to investigate and discuss whether additional knowledge regarding similar ways of classification and multiusage of edible *Alliums* such as shallots/potato onions (*A. cepa* L. Aggregatum-Group), chives (*Allium shoenoprasum* L.) and Welsh onions (*A. fistulosum* L.) can be found, and if traditional names are sometimes linked to knowledge of older cultivation systems.

## Materials and methods

Using a method described as source pluralism by Swedish historian Janken Myrdal, diverse source materials of different kinds are combined with the aim to understand phenomena that are scarcely mentioned in historical records [32]. Data from modern inventories and from questionnaires conducted in the first half of the twentieth century have been studied and compared to horticultural practices described in early Swedish garden literature.

### Garden literature

An intense literature search was performed for references to the cultivation and use of various onions. Cultivation practices and plant names for edible *Alliums* described in Swedish garden literature from 1600 to 1850 were searched for and compared to records from folklife questionnaires and from POM’s 2002–2010 inventory of old vegetable plants. The literature was chosen to include all of the oldest known Swedish garden literature describing cultivation of the studied species, up to and including the most important books published in the second half of the nineteenth century, after which there was a significant increase in the amount of garden literature being published. The same literature has been the focus of earlier studies with a special interest in the practice of planting multiplying onions in autumn [22].

### POM inventory

The current study also draws on documentation collected from surveys carried out by POM where information was gathered from gardeners about old heirloom onions and cultivars.

One of the main findings from POM’s inventory (2002–2010) was that traditional knowledge and practices associated with small-scale gardening for self-sufficiency held by women were often connected to specific heirloom plants [30, 33]. For details, see Strese and de Vahl, 2018 and Svanberg and de Vahl, 2020 [17, 30]. Memories of older relatives were also often tied to specific garden plants [34] (Table 1).

**Table 1:** Cultivations practises and name use of cultivated taxa of *Allium* in Swedish garden literature 1692–1867

Author	Year	Taxonomically understood as	Common name used	Plant date	Harvest date	Onions as sets?	Other	Use
Rålamb [35]	1694	*A. cepa*	Löök	Early spring by seed	When leaves wither	Yes, in spring	Sow together with lettuce, kale and parsnip. The thin onions from Brunswick are better than the ones from Bamberg and Strasbourg	n/a
Rålamb [35]	1694	*A. cepa* Aggregatum Group—shallot	Charlotter	Autumn	When leaves wither	Yes, small onions saved as sets	Can be left in the ground in cultivation for 3–4 years if grown in sandy soil to promote bigger onions. True seed are available from Italy	The large onions are eaten and the small one kept as sets
Rålamb [35]	1694	*Allium* spp.	Kol- eller jacobslök	End of July (as the moon wanes)	In spring for use. Onions for propagation are kept in cultivation until replanting in July	Yes	Cultivated together with lettuce and common cornsalad (Valerianella locusta)	As food in spring
Ahlich [36]	1722	*A. cepa*	Holländsk Röd-Lök	In April (as the moon wanes) as seed	End of July when the leaves wither (after jacobii, as the moon wanes)	n/a	There are white, yellow and red onions, all with the same requirements	n/a
Ahlich [36]	1722	*A. cepa* Aggregatum Group—shallot	Charlotter	End of July (after Jacobi, as the moon wanes)	End of July (by Jacobi, when the leaves wither)	Yes, small onions saved as sets	Can be left in the ground in cultivation for 2–3 years if grown in sandy soil to promote bigger onions. Can be sown as true seed but this is unnecessary when enough small onions are harvested	Large onions are used as food
Ahlich [36]	1722	*Allium* spp.	St Johannis Löök	In August (as the moon wanes, three weeks after harvest)	In August	Yes, onions for propagation can be kept in cultivation until replanting		Can be used as food in May
Dahlman [37]	1728	*A. cepa*	Löök	In spring (as the moon waxes) as seed	Seeds can be harvested if inflorescences are cut off and stored inside to mature in autumn	n/a	There are white, yellow and red kinds. Some gardeners replant the onions in pits when they are pea-sized	n/a
Dahlman [37]	1728	*A. cepa* Aggregatum Group—shallot	Scharlotter	In autumn	When the leaves begin to wither	Yes, small onions saved as sets	Can be left in the ground in cultivation for 3–4 years if grown in good soil to promote bigger onions	Large onions are used for cooking.
Dahlman [37]	1728	*Allium* spp.	Johannis löök	In August (as the moon wanes)	In spring for use. Onions for propagation are kept in cultivation until replanting	Yes		Used as food in spring
Kammecker [38]	1731	*A. cepa*	Holländsk Löök	In April (as the moon wanes) as seed	n/a	n/a		n/a
Kammecker [38]	1731	*A. cepa* Aggregatum Group—shallot	Charlotter	End of July (soon after harvest by Jacobi, as the moon wanes)	End of July (by Jacobi)	Yes, small onions saved as sets	Can be left in the ground in cultivation for 2–3 years if grown in sandy soil to promote bigger onions. Sets are separated and cleaned before replanting	
Kammercker [38]	1731	*Allium* spp.	St Johannis Löök	In August or September (three weeks after harvest date)	In August (as the moon wanes)	Yes		n/a
Broocman [39]	1736	*A. cepa*	Rölök	As seed in early spring	24 August (Bartolomei)	Unclear	Sown together with parsnip, lettuce and anise. Dried before being stored and kept from freezing in winter	Used in several home remedies
Broocman [39]	1736	*A. cepa* Aggregatum Group—shallot	Schalotter	29 September (Michaelmas)	When leaves wither	Yes	A species of onion that doesn't set seed in Sweden	
Lundberg [40]	1780	*A. cepa*	Rödlök (Cepa vulgaris)/ Hwit Spansk Lök/ Gul Holländsk Lök/	By seed in late April–early May. The yellow Dutch and white Spanish varieties can be sown for transplanting in March	When ready, not too late	n/a		n/a
Lundberg [40]	1780	*A. cepa* Aggregatum Group—shallot	Johannis-Lök	In August, replanted every year	Soon after Midsummer	Yes	Same cultivation instructions for St John's onion, shallots and chives (*A. schoenoprasum*), with the exception that chives are to be replanted every three years	n/a
Lundberg [40]	1780	*A. cepa* Aggregatum Group—shallot	Charlotten-Lök	In August, replanted every year	Soon after Midsummer	Yes	Same cultivation instructions for St John's onion, shallots and chives (*A. schoenoprasum*), with the exception that chives are to be replanted every three years	n/a
Fleischer [41]	1795	*A. cepa*	Rödlök	Round varieties: as soon as possible in spring. Elongated varieties: a little later. The yellow and the Spanish red varieties can be sown for transplanting	When leaves are turning yellow	n/a	The known varieties are 'large Italian' (winter hardy), 'common red', 'large yellow' and 'white'. More elongated forms of the white and the common red are also cultivated as 'winter onion'. Sown in spring to be eaten in winter. The common variety in common use is a flat globe shape. The biggest and shiniest onions are selected as seed stock	n/a
Fleischer [41]	1795	*A. cepa* Aggregatum Group—shallot	Scharlott lök (A Ascalonicum)	Preferably in September	In July when leaves are turning yellow	Yes, small onions saved as sets	Contrary to common practice, the leaves shall not be eaten in spring	Large onions are used as food
Jörlin [42]	1796	*A. cepa*	Röd-lök	Early spring by seed	In August	n/a	Stored twined with straw, in a net by a chimney or in dry ash	The Russian or Egyptian variety gives onions the size of small turnips. Pickled in vinegar in stone pots, these will taste rather pleasant
Jörlin [42]	1796	*A. cepa* Aggregatum Group—shallot	Skarlotten lök. *Allium molchatum*	Autumn or spring (as chives)	Replanted in autumn or spring, perennial	Yes	Winter hardy	Can be used in early spring
Ihrström [43]	1808	*A. cepa*	Rödlök	Direct sown in field by seed. For sets: As seeds in end of July	In September. Small onions for sets: harvested end of September or October	Yes, onions the size of nuts are kept as sets. Planted in spring for an early harvest of large onions. Some varieties give many onions per set which increases the yield	Two varieties of common red onion are grown: 'Thick Red' and 'Flat Red'. Also: 'White Spanish', 'White Dutch', 'Yellow Dutch', 'Light Red', 'Portuguese'	In Russia, breeding has resulted in a cross between 'yellow Dutch' and 'light red Portuguese' which form clusters of 3–4 onions per set when replanted
Ihrström [43]	1808	*Allium* spp.	Johannislök	By end of September	August	Yes	Shallots, Welsh onion and St John's onion are three varieties of *Allium fistulosum*. Johannes onion differs from Welsh onion by the withering of its leaves in August, just like shallot	n/a
Ihrström [43]	1808	*A. cepa* Aggregatum Group—shallot	Charlottenlök	In spring	After end of August (like other kinds of onion)	Yes	Shallots, Welsh onion and St John's onion are three varieties of *Allium fistulosum*. Shallots are an early kind of onion that are winter hardy in our climate but never set seed	Gives an early and quite tasty crop
Vothmann [44]	1837	*A. cepa*	Röd-Lök	By seed in early spring, direct sown or for transplanting	When leaves are turning yellow and falling	n/a	Parsnip, lettuce or kale are sown between the rows. Two varieties are known: the large and high 'Hochheimer' and the 'flat globe red onion'	n/a
Vothmann [44]	1837	*A. cepa* Aggregatum Group—shallot	Schalottenlök	October (can be planted in both autumn and spring)	After Midsummer	Yes, small onions used as sets	Spring planting is preferable in humid soils	Large onions are used as food; the leaves can be eaten at spring time
Lundström [45]	1841	*A. cepa*	Rödlök	Both sown in field in spring, and as sets in spring and by seed for transplanting in April	First onions from sets by Midsummer, followed by the sown onions and lastly the transplanted larger onions	Yes, small onions the size of peas and nuts are sorted out in autumn to be used as sets in spring	There are six known varieties (yellow Dutch, yellow Portuguese, red Dutch, red Spanish, white Dutch, white Spanish). Domestic seed are better than foreign	n/a
Lundström [45]	1841	*A. cepa* Aggregatum Group—schalottenlök	Shalotten-lök and johannis-lök	Spring or autumn (same way as garlic)	July	Yes	"Shalotten-lök" and "johannis-lök" are two varieties of same species [*A. ascalonicum*] and resemble each other	n/a
Muller [46]	1850	*A. cepa*	Rödlök	By seed in spring and for transplanting in March or April. By sets in spring	When leaves are turning yellow. Onions from sets can be harvested soon after Midsummer	Yes, small onions kept from last year's harvest are used as sets and produce large onions early. These cannot be stored well	Many varieties are known. Varieties meant to be small in size ('Dutch pearl onion') are sown more densely	n/a
Muller [46]	1850	*A. cepa* Aggregatum Group—potato onion/Nordic shallot	Nordiska Schalotten, *Allium* cepoides	Early spring	Autumn	Yes	The onions are yellow and the size of walnuts. Gives a rich and reliable yield. Are winter hardy and stored like red onion	Tastes like red onion but is superior
Muller [46]	1850	*A. cepa* Aggregatum Group—shallot	Schalotten, *Allium escalonicum*	Early spring	Autumn	Yes	Comes from Palestine	n/a
Anderson, J.F. [47]	1852	*A. cepa*	Rödlök	By seed in spring and for transplanting into pots or containers. By sets in spring	As for garlic: when leaves are turning yellow the length of one finger from the top	Yes, small onions the size of hazelnuts are kept from last year's harvest and used as sets, producing good onions soon after Midsummer	Many varieties are known	n/a
Anderson, J.F. [47]	1852	*A. cepa* Aggregatum Group—potatislök/nordisk schalottenlök	Nordiska Schalottenlöken	Can be planted in autumn for a bigger and richer yield	When ready after Midsummer (as garlic)	Yes, propagated by small onions formed around the large ones	Winter hardy	n/a
Eneroth [48]	1867	*A. cepa*	Rödlök	By seed in spring or by sets in early spring	When leaftips are turning yellow. Sets are harvested in June	Yes, small onions are kept from last year's harvest and used as sets, producing large onions early. These cannot be stored well	The variety 'white Dutch onion' also known as "pearl onion" [syltlök] is less commonly grown by poorer households. The best varieties are 'blood red Dutch' and 'Strasbourger'	n/a
Eneroth [48]	1867	*A. cepa* Aggregatum Group—potato onion/Nordic shallot	Rysk Scahlottenlök [Russian shallot]	Autumn, simultaneously with the harvest	Autumn	Yes, small onions saved as sets	Can be grown in the far north of Sweden	The flesh colour resembles the 'White Dutch Onion' but the other parts are light brown. Smells like Red Onion but cloves are irregular in shape since it grows in clusters
Eneroth [48]	1867	*A. cepa* Aggregatum Group—shallot	Vanliga Schalottenlöken [Common shallot]	Spring	Autumn	Yes, small onions saved as sets	Are only suitable for the south of Sweden	Are tastier but not as large as the Russian shallot and not as white in the flesh

Green coloured rows are interpreted as *A. cepa* L., orange as *A. cepa* L. Aggregatum-Group, white as unidentified *Allium* spp. and yellow as a certain cultivar or subspecies of shallots well adapted for Nordic growing conditions

### Digitised questionnaires

Fully digitised folklife (i.e. ethnographic) questionnaires from the Nordic Museum were searched for records mentioning species of onion, garlic, chives and leek, all including the Swedish common name for onion (*lök*) in their common names. These records were categorised according to the different uses and practices described for the various species and labelled by region. The manuscripts at the Lund University Folklife Archives are digitised but only searchable for index words. Records indexed with the species names in Swedish were included in the study, but since these results in relation to gardening practices and cooking were relatively few and regionally very unbalanced for onion, they were excluded from Tables 2, 3 and 4, and were instead used to fill in the picture of the extended use of these plants in folk medicine and traditions.

**Table 2:** Use of *A. cepa* L. in food preparation from Nordic Museum questionnaires

Sausage	46
Pölsa/brawns/offal	18
Meat dishes (festive)	18
Spread and farinaceous foods	18
Potato dishes	16
Soups	16
Other	15
Herring—pickled	13
Fish	13
Herring—fried	12
Pickled onions	11
Herring—others	9
Palt (potato dumplings)	8
Pork and meat (everyday dishes)	7
Kroppkakor (potato dumplings)	6
Black pudding	6
Caviar	6
Bird dishes	6

**Table 3:** Use of *A. schoenoprasum* L. in food preparation from Nordic Museum questionnaires

Fish	14
Spread	10
Sauce	6
Other	6
Meat	4

**Table 4:** Use of *A. ampeloprasum* L. Porrum-Group in food preparation from Nordic Museum questionnaires

Soup	10
Meat	4
Other	3

Source criticism of the archive material collected and curated in the first half of the twentieth century, has pointed out that women’s perspectives, as well as those of the less wealthy classes of the rural societies, were excluded. Ethnologist Agneta Lilja argues that certain geographical areas were given priority by the archives while others were rejected as a reflection of scientific biases and views of the male farmer as a lost ideal [49]. Later research has suggested that the source material held by the folklife archives might still contain valuable and diverse knowledge, even though the political debates and struggle tied to a growing labour movement is reflected in the collections [50]. Historian Fredrik Skott has shown that the questionnaire respondents in the same material often did in fact represent a variety of professions at the time well among the men, and also included representation of both old women and men on the whole [50]. Studies of food culture have revealed problems with records from certain regions gathered via Nordic Museum questionnaires regarding sausage recipes being completely absent from the material, while others are overrepresented [51]. When searching for records of the use of onions in the Nordic Museum’s collection ‘Nm 3—Food preparation and meal customs’, it was likewise obvious that the provinces of Scania, Småland and Dalecarlia were overrepresented (together representing 48% of the records studied) in the material, while regions of northern Sweden were underrepresented (with Jämtland as an exception) (Fig. 6). The lack of records for certain cultivated species grown and lack of information regarding cultivation practices, inheritance and/or trade of plant material of the peasant gardens may be explained by the absence of questions on these matters from the interviews.

## Results

### Cultivation practices of onion,* A. cepa*

#### Autumn planting and true seed

Older cultural descriptions from the reviewed literature are presented in Table 1. The table shows that autumn planting of both shallots and the more hard-to-identify cultural forms of onions called *Johannislök* (‘Saint John’s onion’) and *Jacobslök* (‘Saint Jacob’s onion’) is advocated until the mid-nineteenth century, while the earliest descriptions of a certain kind of shallot especially suitable for northern conditions appear in the 1850s.

The moon is frequently referenced as an indicator for planting and harvest dates, in the same time as the names of saints are often used in the literature. The authors are inconsistent regarding whether the onions should be planted by the waxing or waning of the moon, which corresponds to other records from questionnaires on this subject. Several records from Scania and Småland state that onions shall be planted at the waning moon, while the opposite instructions are given in the province of Halland in south-western Sweden [52–54].

The only occurrence of shallot being described as seed-propagated comes from Åke Rålamb in 1694, stating that true seed were available from Italy, and also that shallots could be left in the ground for several years to promote bigger onions [35].

#### Saint John’s onion planted for early harvest

The category of names in Table 1 associated with plant or harvest date given for the unknown species (labelled *Allium* spp.) before 1780 probably refers to Welsh onion, *A. fistulosum* L. The name variants that come from Christian calendar saints such as Saint Jacob and Saint John represent a practical way of naming cultivated plants according to the time of harvest, or to their use. The prefix *Johannes* (i.e. Saint John) is common in many other species names and the shift of name use over time may be explained by the change of calendar system during the period under study (Table 1) [40]. In Sweden, the Julian calendar was gradually replaced by the Gregorian during the eighteenth century, making old popular knowledge connected to certain dates harder to rely upon.

In growing instructions written between 1780 and 1841, several authors do not distinguish between chives (*A. schoenoprasum*), Welsh onion (*A. fistolosum*) and shallots (*A. cepa* L. Aggregatum-Group) [40, 43, 45]. Later, authors use the name *Johanneslök* as an synonym for shallots [55, 56].

Linnaeus refers to the onion eaten in church as the ‘true’ (*rätta*) Saint John’s onion when describing it in the province of Dalecarlia in the 1730s, but in other sources he describes it as differing only *in gradu* from *A. fistulosum.* In other records, he also identifies it under the common name for *A. schoenoprasum* var. *sibiricum* (L.) Hartm. This indicates that the taxonomic belonging and identity of this onion might have been in question even then.

#### Miller’s true scallion

In England, a similar search for an old variety is described by the gardener and botanist Philip Miller using partly the same Latin names as Linnaeus. In 1735, Miller distinguishes between chives, Welsh onion (both given Latin phrase name derived from *Cepa sectilis),* scallions (*Cepa ascalonicum*) and ciboule (*Cepa fisfillis*) [57]. None of these are described in the text as what we know today as shallots, but were eaten in early spring as green crops. Miller describes how the scallion at the time was known only from botanical gardens and had been replaced by a new onion without winter rest that was sold in bunches in spring as scallions.

When Miller’s text was partly translated into German by Franz Hermann Heinrich Lueder in 1780, the old onion no longer in cultivation was identified as the crop he calls *Johannislauch* and classified with the Linnaean binominal name as *A. schoenoprasum* L. [58].

In the late nineteenth century, the name variant *Johanniszwiebeln* was used in parallel with *Johannislauch* in Germany. An article in the magazine *Allgemeine deutsche Garten-Zeitung* in 1823 divides onions into the categories *Sommerzwiebeln* (‘summer onion’), *Johannis*-*Zwiebeln* (‘St John’s onion’), *Saz-/Stekzwiebeln* (‘Saz onion/frying onion’), *Winterzwiebeln* ‘winter onion’ and *Perllauch* (‘pearl onion’) [59]. The Saint John’s onion is described not as a particular species or cultivar but as a crop, sown on Saint John’s Day, *johanni,* for an early harvest the following spring. It is also stated to be increasingly rare in cultivation since the winter onion and pearl onion could be cultivated in the same way.

Questionnaires and newspaper reports confirming the existence of this cultivation system in Sweden are rare, but show a continuity in an old Nordic tradition of harvesting onions for Midsummer [60]. From the province of Värmland, a corresponding author writes about *Pehrsmässolök* or *Johannislök* in the magazine *Inrikes Tidningar* 1741. The onion is planted on Saint Lawrence’s Day (10 August) to be harvested the following summer on Saint Peter’s Day (26 June). The crop is praised by the author in Värmland for giving an early yield at a time of food shortage [61]. In a questionnaire response from Dalecarlia, the name *vårlök* (‘spring onion’) is used for onions planted at Midsummer and harvested in the autumn [62].

In Denmark, the related name *Sankt Hansløg* is used from the eighteenth century; in south-west Jutland, *midsommerløg* was a folk name known from 1860 [63].

The use of common names referring to Midsummer (24 June, the feast day of John the Baptist in the Christian calendar) for a crop producing an early harvest was maintained in the first half of the twentieth century, when a certain product of *A. cepa* L. treated to work as sets for an early harvest was introduced as *mi’sommarlök* (‘Midsummer onion’) or *snabblök* (‘swift onion’). This was marketed as superior to the old practice of saving small onions as sets and involved treated onions of already known cultivars such as '*Zittauer'* and '*Braunschweiger'* [64].

The fact that autumn planting is not documented in the questionnaire responses is coherent with the fact that such cultivation instructions were less consistent in the latter part of the nineteenth century (Table 1).

#### The moon and the calendar

The authors of the garden literature presented in Table 1 are inconsistent on whether the onions shall be planted at the waxing or waning of the moon, which corresponds to other records from questionnaires on this subject. Several records from Scania and Småland state that onions shall be planted at the waning of the moon, while the opposite instructions are given in Halland [52–54]. In answers to a questionnaire about sowing times, respondents recall practices from the final decades of the nineteenth century in Småland involving the use of the calendar in relation to the moon. A respondent from Kråksmåla parish says: ‘The old ones said, “A sane man sows grain, peas, onions and beans at the waxing of the moon.” NB! The waxing of the moon did not always work with the timing of the spring sowing, when it is related to *Suge* ('week-counting')’ [65].

Another respondent from Småland explains the tradition of week counting, called *vekeräkning*, in relation to the writings of Carl Linnaeus on the matter during his Scanian tour in 1749. Linnaeus describes how Scanian peasants counted the weeks backwards in the spring in relation to Vårfrudagen (Annunciation Day) and Midsummer’s Day to determine the time for sowing [66]. The respondent explains how this practice was still used by his father in the 1880s and that week number 13 corresponded to the former holiday date of 6 April, counting down to week 1 ending on 6 July [67]. The change of calendar during the eighteenth century explains why the holidays could no longer be used as indicators of tasks in the annual work-cycle. The practice of *vekeräkning* probably became a more reliable and functional indicator than the waxing and waning of the moon.

While beliefs and practices connected to the moon were not encountered during the POM inventory taken in the early 2000s, knowledge and traditions related to the almanac were described in accounts from Dalecarlia. One example is the tradition of sowing multiplying onion sets at Ersmäss, the Feast of Saint Erik, on 18 May and harvesting them on Saint Bartholomew's Day on 24 August, which is still practised by the local community in Leksand [18, 68].

#### Pickled or used as pearl onion

The old traditional way of using small specimens of common onion (*A. cepa* L.) as sets the following spring is first mentioned in growing instructions given by Åke Rålamb as early as 1694 [35]. This practice is then not found in the literature until 1808, when gardener Carl Ihrström describes how onions the size of nuts are kept as sets and planted in spring to give an early harvest of large onions [50]. He also states that certain varieties produce many onions per set, which increases the yield, and that certain Russian cultivars with this quality have been produced by crossing different European cultivars. The tendency to form many small onions in one cluster is described by Ihrström as a desirable quality. Descriptions of the practice are to be found in all the literature studied, together with the consistent statement that onions grown in this manner are not storable. In 1795, botanist Engelbert Jörlin writes about how the Russian and Egyptian varieties produce onions the size of small turnips that can be pickled in vinegar in stone pots and taste rather pleasant [42]. Some early instructions for using heat treatment to encourage more growth and fewer flowers are found in German literature translated into Swedish in the early nineteenth century [69]. Onions are to be stored near a hot oven for the winter to increase in size over the coming season.

Certain cultivars described as pearl onions (*syltlök*) are seldom found in the literature studied, but Müller in 1850 explains that the 'Dutch pearl onion' should be sown more densely since the onions are supposed to be small [46], and Eneroth states in 1867 that the variety 'white Dutch onion’ also known as ‘pearl onion’ (*syltlök*) is less commonly grown by poorer households [48].

These literature records and cultural descriptions of common seed-propagated onions of *A. cepa* L. provide clues to the early practice of a cultivation system whereby small bulbs were saved over the winter and used as sets to give an early harvest, and the emergence of pickling onion (also translated as *syltlök*) as a cultivation system that may have preceded the introduction and knowledge of specific cultivars adapted to be grown in this manner.

In the Nordic Museum questionnaires, there are relatively few mentions of growing practices regarding pickling onion, but several records of recipes indicating that pickling onions were known and often prepared with nutmeg at the turn of the nineteenth century, at least in south of Sweden (Table 2). In two of these records, the term ‘white onion’ or ‘small white Spanish onion’ is used to refer to the kind of onions used as pickling onions [70, 71].

There are records of pickling-onion cultivation from Uppland [72] and Dalsland [73] but also from Västergötland, where the respondent speaks about his father travelling in to the city of Gothenburg to sell pickling onions sorted into different sizes in the town square [74]. Sorting the onion harvest by size is also reported from Scania, where the common onion (called *rödlök*) was kept in storage layered with birch ashes. The small onions were stored at the bottom, since they had higher durability in storage than the large ones. After damp summers, the large onions were not stored at all. To keep them from mould, they were instead diced, dried and kept in glass jars to be used for steaks and sauces during the winter.

Records indicating that ‘pickling onion’ (*syltlök*) and ‘small white onion’ were common names used to distinguish the product of the harvest according to use in the kitchen rather than to taxonomy, correspond to earlier findings that these names have sometimes also been used as common names for shallots or potato onions [22].

#### Cultivation practices

Mentions of onion are frequently found in the data from the Nordic Museum questionnaires ‘Nm 3—Food preparation and meal customs’ and ‘Sp 111—Spices’. The study of these records revealed that onions were a natural part of Swedish rural garden culture and part of subsistence production for small-scale households. Onions (such as *lök* or *rödlök*, ‘red onion’) are described as frequently cultivated together with carrots, different kind of kale (*Brassica oleracea* L. cvs*.*), peas (*Pisum sativum* L. cvs.), beans (*Phaseolus vulgaris* L. cvs., *Vicia faba* L. cvs*.*)*,* and root vegetables such as red beetroots (*Beta vulgaris* L. cvs*.*), swedes (*Brassica napus* L. Napobrassica-Group), parsnips (*Pastinaca sativa* L.), and sometimes potatoes (*Solanum tuberosum* L.), throughout the country. There are disparities in how the growing sites in the garden are defined, but also in how the crop is categorized. Onions are sometimes grown and categorised as a vegetable and sometimes as a spice, which is also reflected in the double use of the harvest product in the kitchen. In accounts from the Västerbotten region in the north of Sweden, onion is described as the most noble spice and as commonly cultivated even though kitchen gardens were rare in the area [75].

Accounts from Dalecarlia explain how onions were part of a crop-rotation system with swedes and carrots in the beds of the kitchen garden where herbs as marjoram (*Origanum majorana* L.), thyme (*Thymus vulgaris* L.)*,* horseradish (*Armoracia rusticana* G. Gaertn., B. Mey & Scherb*.*), and dill (*Anethum graveolens* L.) were also common [76]. The most common herbs grown and used besides onion according to the questionnaires in general were caraway (*Carum carvi* L*.*), dill, horseradish, parsley (*Petroselinum crispum* (Mill*.*) Fuss), anise, (*Pimpinella anisum* L.), marjoram and thyme.

When onions are described to be grown as a spice, chives (*A. schoenoprasum* L.) are often also mentioned as well, but since the Swedish common names resemble each other, it is sometimes unclear whether chives are included in the descriptions of onions or not.

An account from Värmland states that potatoes, kales and swedes all had their own specific growing fields while, e.g. onions, carrots and dill were grown in the kitchen garden [77]. Since the kale field required more nutrition, it was fertilised with manure from outdoor toilets, while cow and horse manure was used for the other garden crops. This was a task mainly for the women and something that the men preferred not to deal with, according to the respondent. According to the folklife questionnaires in general, potato was the most common crop, but since it was grown as a field crop it was more seldom also grown in the kitchen garden.

The practice of cracking the stalks of the onions (*luka löken*) before harvest is described by one respondent, a former gardener, Frans August Gustavsson from Småland, who also states that this use of red onions (*rödlök*) is hardly worth mentioning since it is commonly known in both the cities and the countryside [78]. Olof Christoffersson also describes the practice in his cultural-historical description of Skytts härad (‘hundred’ an administrative unit larger than parish) in the southern region of Scania [79]. Here, the onion stalks needed to be trampled down in silence on Midsummer evening, otherwise there would be no harvest. This practice was also encountered in POM’s inventory taken in the early 2000s, described by various donors from childhood memories of the harvest of heirloom multiplying onions that were kept within the family (*A. cepa* L. Aggregatum-Group) [18].

#### Heirloom varieties of multiplying onion

**Fig. 1:**
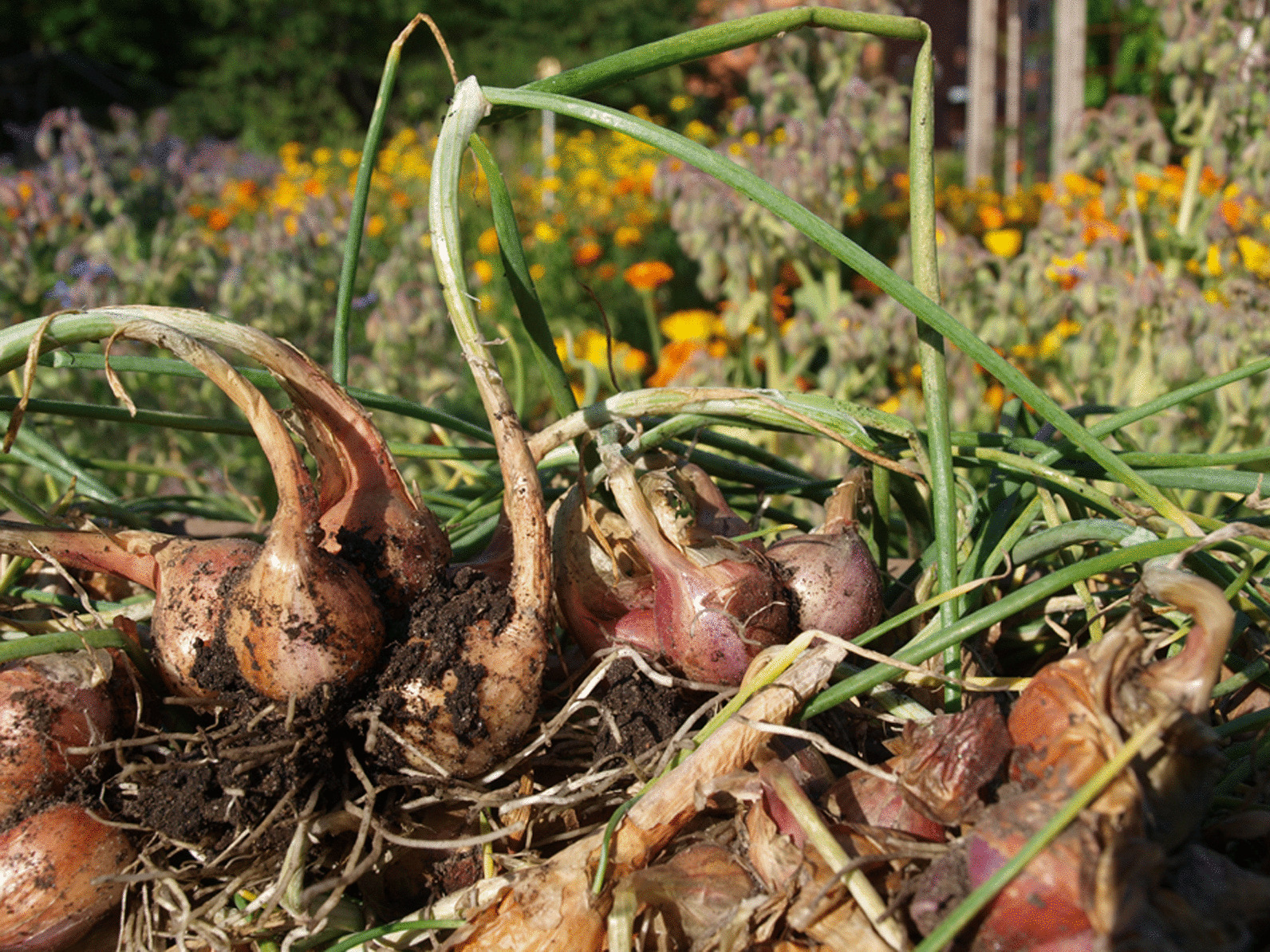
*A. cepa* L. Aggregatum-Group ‘Anna’. One of many heirloom varieties collected from Dalecarlia Photo: Erik de Vahl

From the north of Sweden, there are written records of onions being dried and stored hanging from the ceiling in Jämtland [80], and kept in fish trays by the ceiling in Hälsingland [79]. In POM’s inventory, the storage capacity of the old heirloom varieties of multiplying onions (Fig. 1) was one of the major qualities praised by donors as superior to that of modern varieties [18].

A respondent from Värmland states that the crop known and described above as onions (*lök*) were shallots (*A. cepa* L. Aggregatum-Group). They were vegetatively propagated and shared around by neighbours if the yield failed. Once the crop had started to grow a little, the rows were earthed up [77].

From Dalecarlia Värmland and Scania there are records stating that the most commonly grown onion was multiplying onion, known in Scania as potato onion (*panntofflalög*, *pärelög* or *potatislök*) [81], in Dalecarlia as *Leksandslök* [82] and in Värmland as shallots (*chärlottlök* or *chalottlök*) [83].

#### Name use of red and white onion

It is clear that the term ‘onion’ (*lök*) may include many different cultigens known to the respondents, and should preferably be interpreted as referring to the culton believed by the respondent to be the most common in Tables 2, 3, 4 and 5.

**Table 5:** Use of *A. sativum* L. from Folklivsarkivet and Nordic Museum questionnaires

Human protection against evil	75
Human medicine	30
Human use (other)	9
Livestock medicine	18
Livestock protection	18
Livestock in between	6

**Fig. 2:**
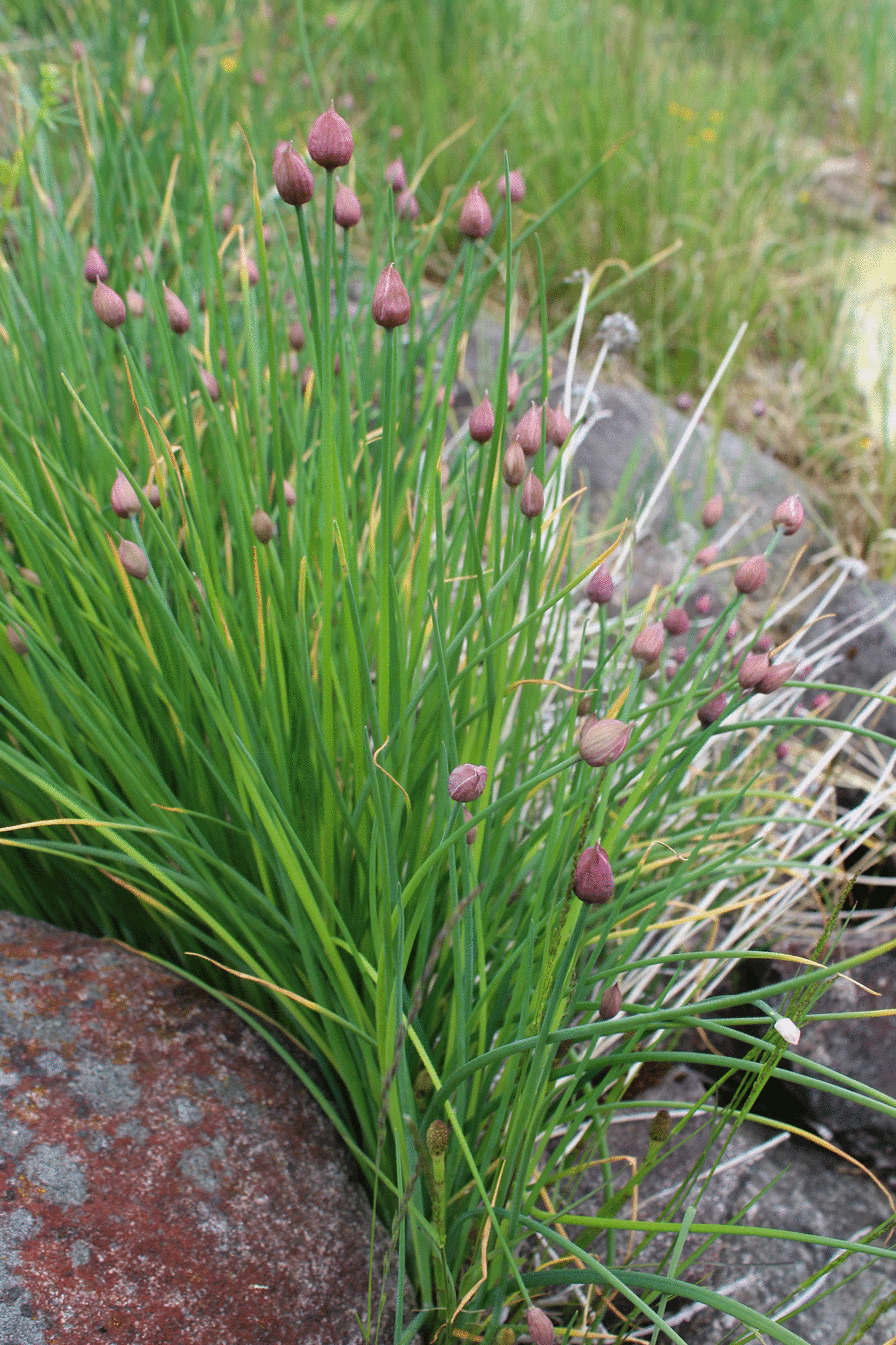
*A. schoenoprasum* L., Chives growing by the lake in Rällsjöbo Dalarna. Photo: Erik de Vahl

**Fig. 3:**
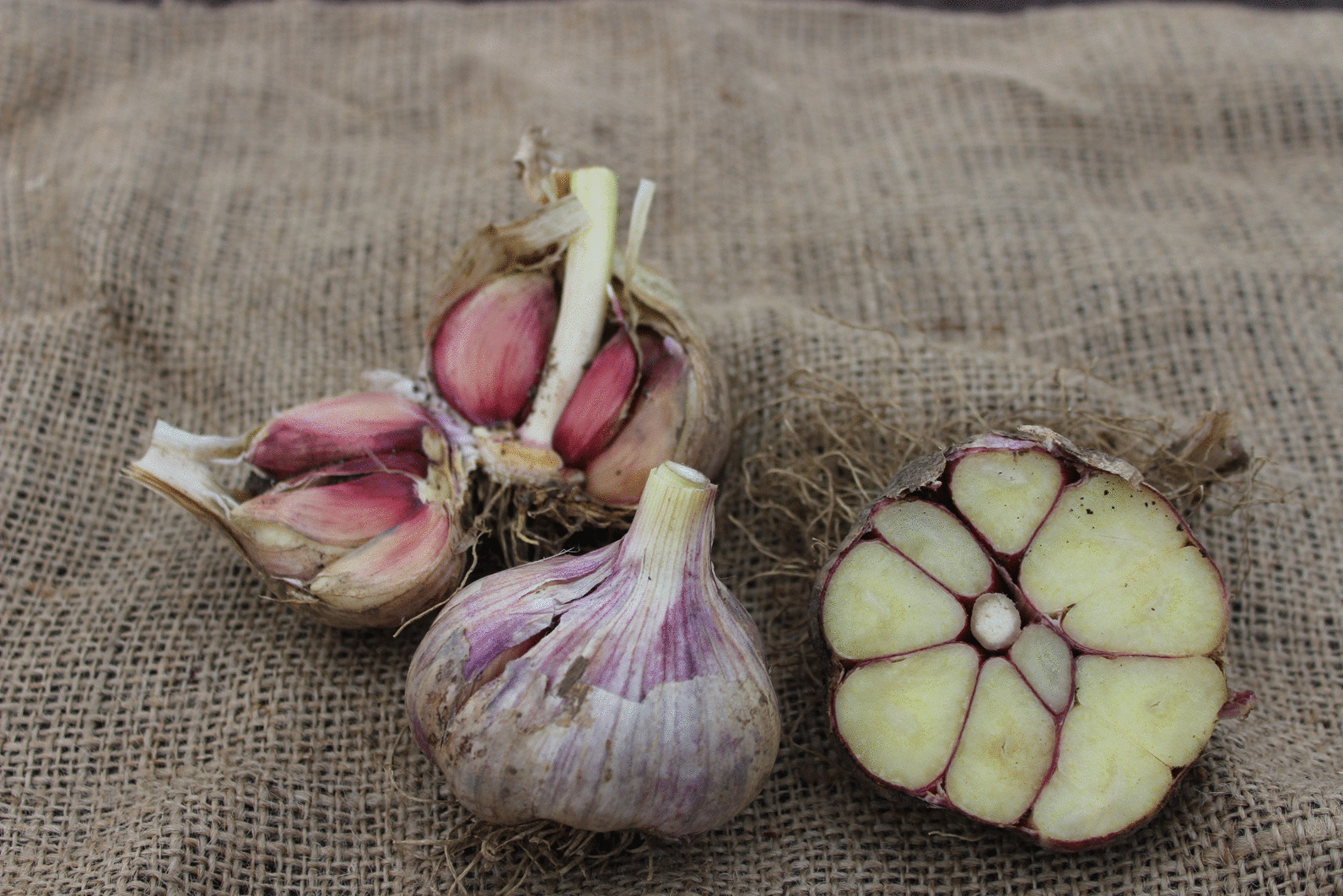
*A. sativum* L. ‘Gusum’. Garlic collected in POM’s inventories and preserved at the National Gene Bank for Vegetatively Propagated Crops in Alnarp. Photo: Erik de Vahl

The inconsistent Swedish tradition of using the common name ‘red onion’ (*rödlök*) as a collective term for the taxa *A. cepa* L. as an opposite to garlic, *A. sativum* L., in Swedish called ‘white onion’ (*vitlök*), is reflected in the records from the questionnaires (Figs. 2, 3).

**Fig. 4:**
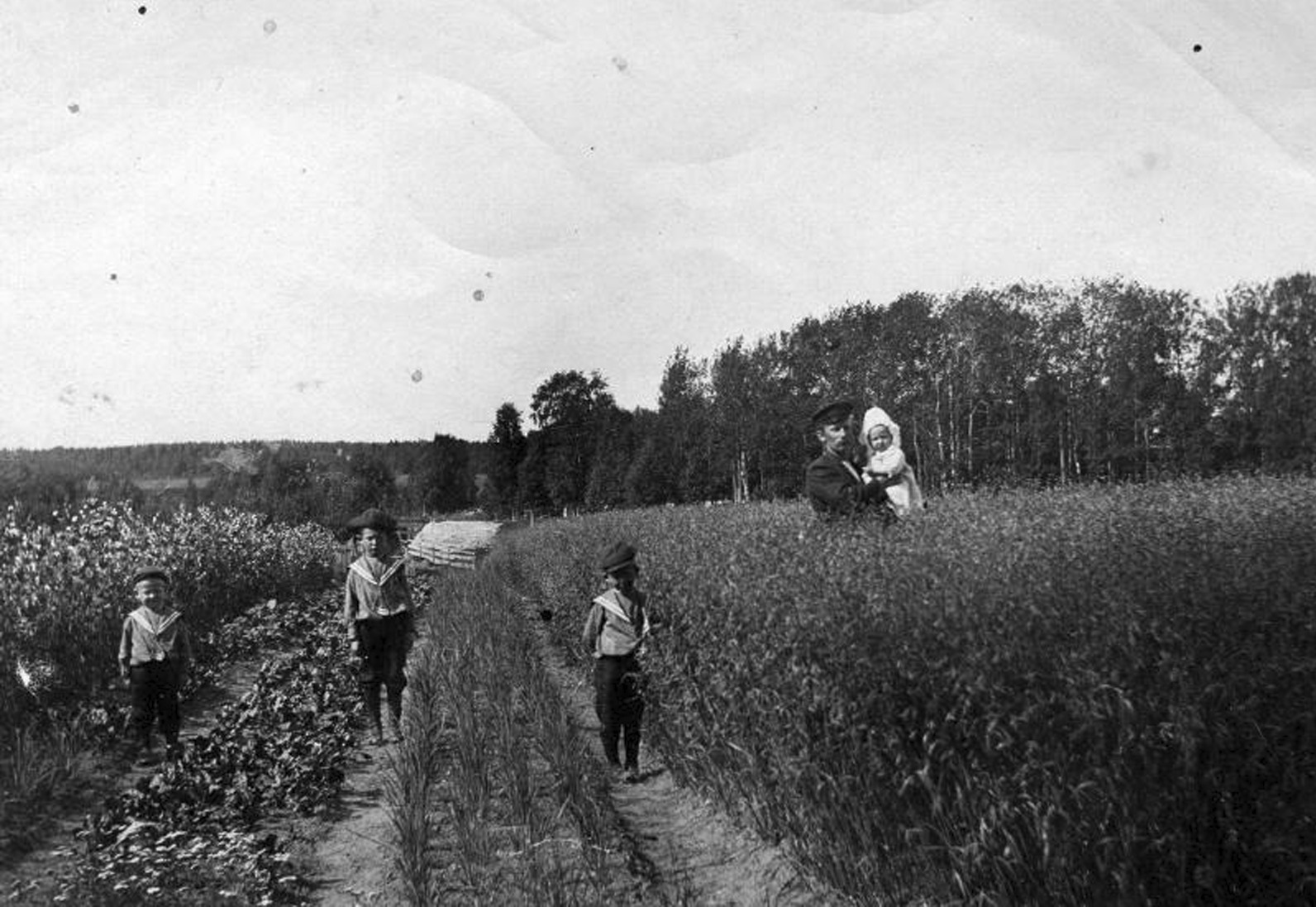
Onion cultivation in Mjöfjärden in Norrbotten, early 1900s. Photo: Agabus Björk, föreningen raan.nu

In the northern regions of Sweden, the term ‘red onion’ is not used at all, while it is frequently used in Scania, Småland, Värmland and Dalecarlia in the south, either to distinguish between multiplying onions and seed-propagated onions or between onions and chives. One respondent from Dalecarlia mentions the red, the white, and the common onion, where the latter must be understood as the multiplying onion known in Dalecarlia as *Leksandslök* [84]. In addition to literature records stating the shallot to be the onion most commonly cultivated in the north of Sweden by the turn of the nineteenth century [85, 86], the records in the questionnaires reinforce the notion that the term ‘onion’ often refers to multiplying onion when used in a popular context (Fig. 4). The POM inventory collected heirloom varieties of multiplying onions from most of the Swedish regions for ex situ preservation, and the colour of the skin and flesh of these varieties range from white to violet.

The name ‘yellow onion’ (*gul lök*) is seldom referenced in the questionnaires but is represented in a few records in other sources and is also referred to by popular cultivar names such as ‘Portuguese onion’ [87].

Earlier studies have shown that Swedish trade, in contrast to garden and botanical literature, had a more rational way of naming early cultivars of onion (*A. cepa* L.) by the colour of their skin, and their origin [22]. Since garlic (*A. sativum* L.) was not used for food seasoning, but only as a medicinal herb until the nineteenth century, it is usually easy to determine whether historical records of white onions refer to garlic or varieties of *A. cepa* L.

### Uses of* Allium cepa* L.

#### Onions eaten in church

Earlier studies have described the tradition of bringing fragrant herbs to church in a bouquet [29] and the related practices of bringing chewed tobacco, resin, snuff or onions to consume during the Mass [17]. The peasantry grew the onions themselves and in summer they were sold in front of the church on Sundays before the service. The custom of eating onions in church was described by several observers, and was sometimes condemned as barbaric and the smell hard to bear. Still, the women seem to have appreciated the taste and the aroma. Several Swedish authors have given accounts of this custom in the region of Dalecarlia, the first being Carl Linnaeus in 1734 [16]. While travelling through the province in summer, he referred to the onions eaten in the church in Bjursås parish as *hvitlök* (‘white onion’) and in Älvdalen parish as *Johannislök* (‘Saint John’s onion’). Hvitlök might have been a local common name for *A. cepa* L., while it is more difficult to be sure of the referent of Johannislök.

**Fig. 5:**
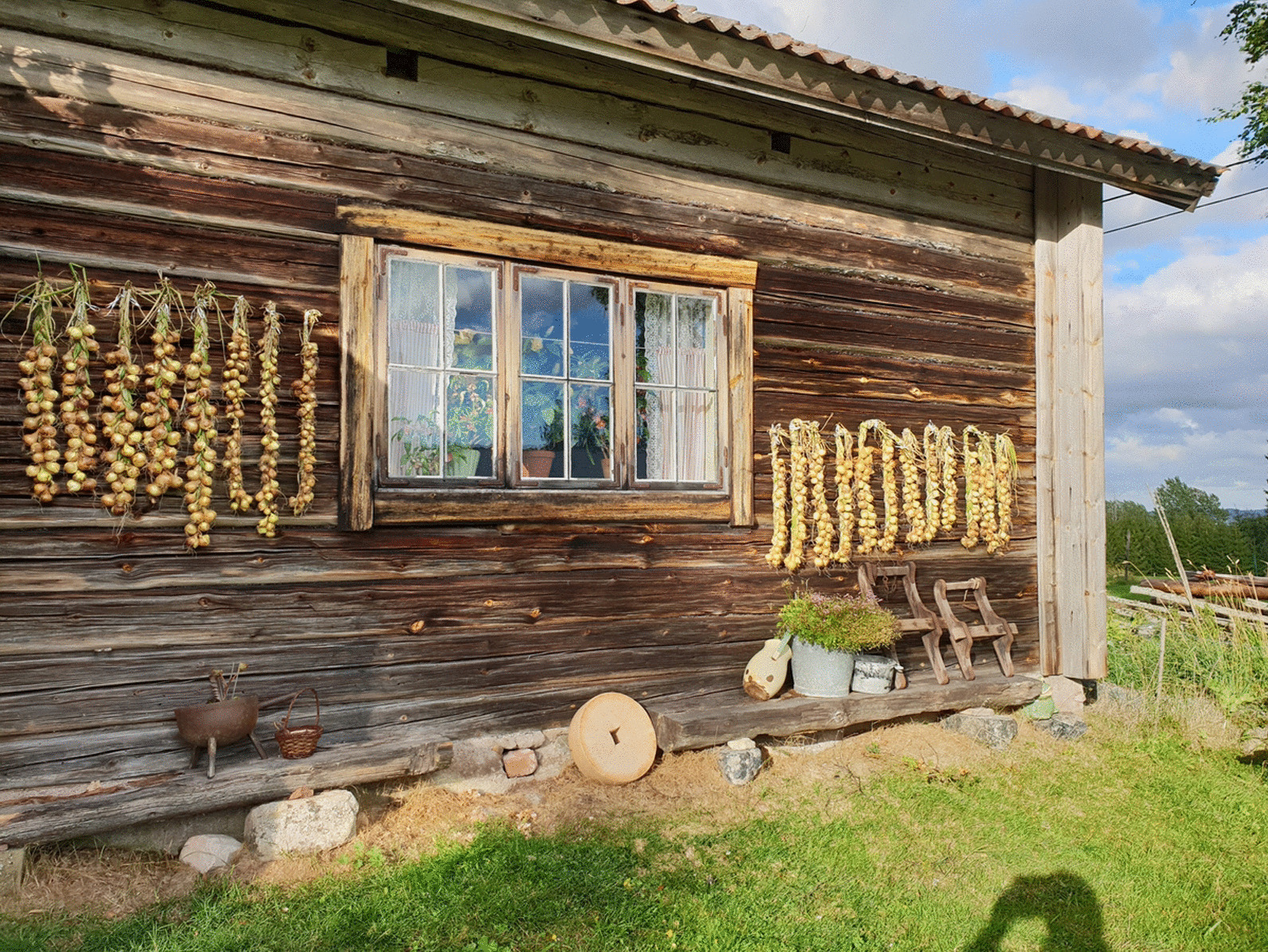
Onions drying on the outer wall of a shieling (mountain hut). Kättboåsen, Venjan, Mora, 2018. Photo: Jennie Tiderman-Österberg, Dalarnas Museum

**Fig. 6:**
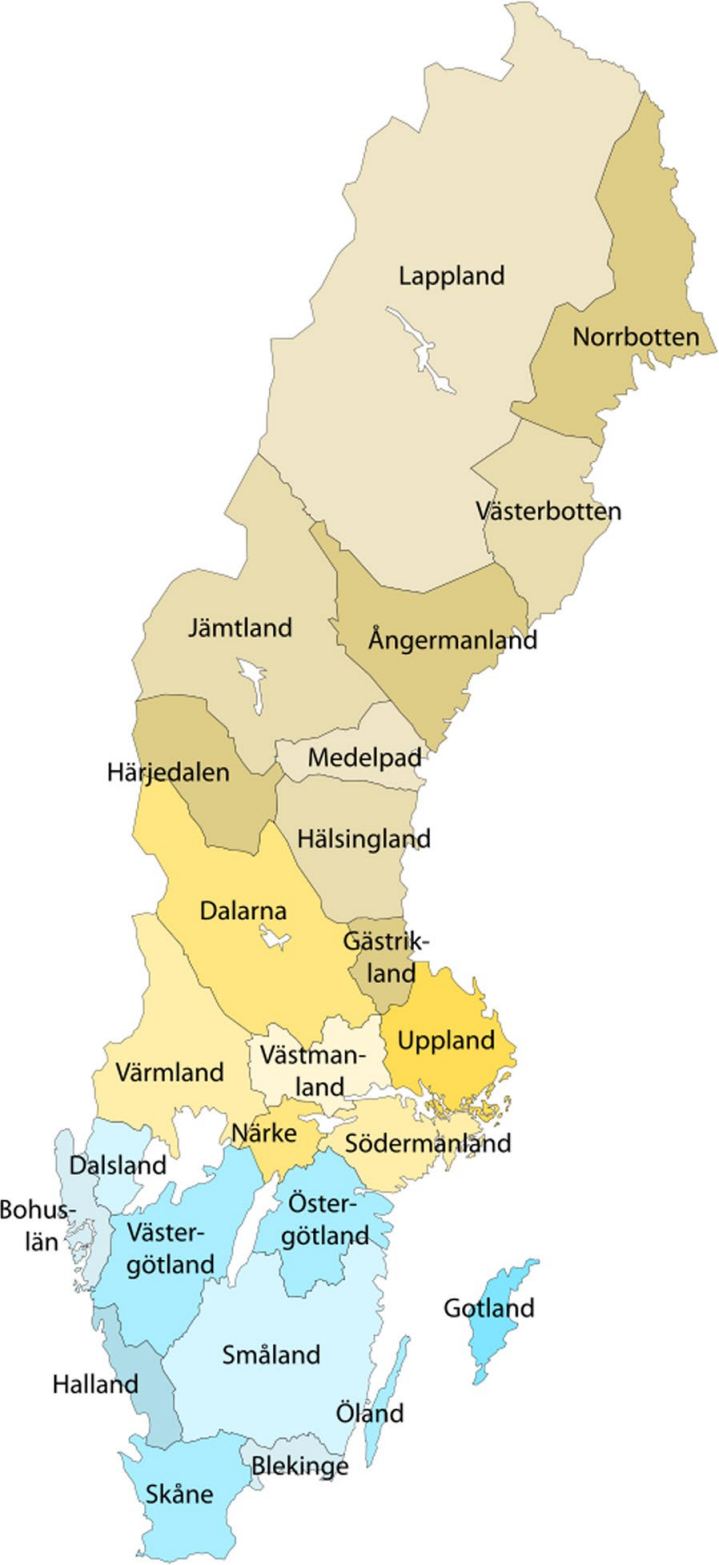
Swedish provinces. Photo: Wikimedia Commons

Other travellers to the region describe the onions eaten in church as ‘white onions of a small kind’ in 1829 [88], as ‘Spanish onions’ in 1859 [89] and as ‘blindingly white’ and particular to the region in 1863 [90] (Figs. 5, 6).

**Fig. 7:**
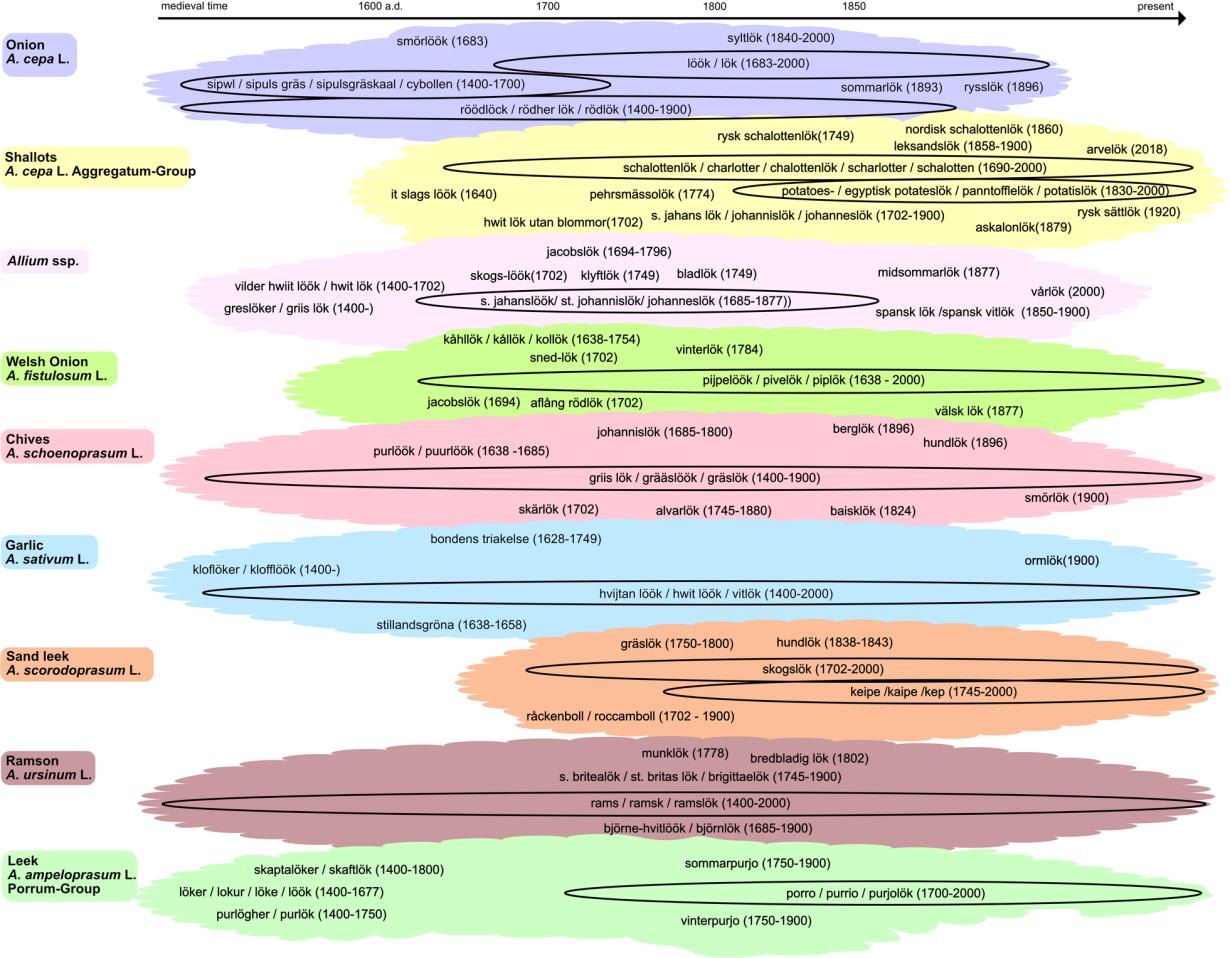
Records of Swedish common names used for cultivated taxa of *Allium* based on Lyttkens [91], Larsson [31], Svanberg and de Vahl [17] and de Vahl [22]. The earliest records refer to medieval documents. They are hard to date but consistently marked as 1400- in the figure. Records marked as 2000 indicate that the names are still in use. Some names were in use for long periods in time

It is fair to assume that different culta (cultivar groups) in the genus *Allium* have been used in similar ways at different times and during different seasons. Linnaeus’ inconsistent use of the Swedish common name *Johanneslök* (Fig. 7), and the double use of the term ‘white onion’ for both garlic (*A. sativum* L.) and culta of *A. cepa*, L. makes interpretation of the source material difficult. Frequent discoveries of heirloom varieties of multiplying onion in the region point to this crop being one of the onions that were often eaten in church.

#### Slaughter food

In peasant kitchens, onion is known to have been used to season soups, blood sausages, dumplings (Swedish *kroppkakor*, *kams*) and various meat dishes [1]. According to the questionnaires in the Nm series, this was the most common use of onion in rural households in the late nineteenth century. It was a common ingredient in different kinds of home-made sausages (Table 2). Various detailed recipes are documented from many different regions. Both red and white onions were used, although the distinction is seldom made in the recipes. Blood sausage is the most common, but cereal sausage, potato sausage and offal sausage (often called *hackekorv*) are also commonly referenced. Together with the Swedish traditional dish *pölsa*, also frequently described as a use for onion, the making of sausage and other charcuteries represents rural knowledge and practices related to small-scale farming and self-sufficiency in pre-industrialised rural communities. From southern Sweden, there are several records of the use of onion in recipes for black pudding and *palt* made from offal (Table 2). The term *palt* often refers to a traditional northern dish of potato, cereals and pork, but the term has historically been used in many Swedish regions with different meanings [1].

#### Onions and herring

Records from questionnaire Nm 145, which asks specifically about fish, show that onions were frequently used as an ingredient in pickled herring, often prepared and served at festivities, but also as an ingredient for everyday cooking in sauces made for fried salted herring (Table 2). The use of onions in recipes for caviar made of northern pike is reported from both the north and south of Sweden.

#### Spreads and sauces

Onions were traditionally used for flavouring butter, lard or grease for use as a spread eaten on bread. This practice is recorded across a wide area ranging from Scania in the south to Ångermanland and Jämtland in the north.

In terms of staple dishes with meat, the most common use of onion was as an ingredient in a sauce made for pork, sometimes called *doppa*.

In southern Sweden, onion is also a common ingredient in everyday soups, such as the factory workers’ soup described in Småland [92]. The respondent describes a root-vegetable soup made with onions, carrots and parsnips as one of three main dishes in rotation in the diet of proletarianised farmers and factory workers in Bankeryd, Småland, in the early decades of the twentieth century.

From Dalecarlia and Jämtland, there are recipes where shallots or *Leksandslök* are ingredients in festive dishes made with meat, and from Scania there are several recipes where onions are used in dishes of goose, chicken pudding and pate, also understood as festive dishes.

#### Dyeing of Easter eggs and medicine

Onion skin has traditionally been used for dyeing eggshells in southern Sweden. Shallot and red onion in particular have been used for that purpose [93]. In the questionnaires from the Folklife Archives at Lund University, there are records showing extensive use of onions in traditional medicine, folklore and dyeing. From Scania and neighbouring regions in southern Sweden, there are several descriptions of onion skin being used for dyeing eggs at Easter. The skins together with moss gave the eggs a yellow colour when cooked. One respondent born in Scania in 1879 describes how the domestic staff of a household were given between eight and 15 cooked eggs each at Easter, depending on the generosity of the master [94]. He remembers the eggs being coloured yellow by onion skins or brown by moss and coffee grounds, and patterned by tying leaves of cow parsley, *Anthriscus sylvestris* (L.) Hoffm., around them. Ears (i.e. grain-bearing tip part) of barley and rye in the tradition of using shallot skins have been described from the region in earlier studies [2]. Onion is known to have been widely used in folk medicine for a number of diseases and illnesses such as cough, gout, scurvy, chills and tapeworms [2].

As with garlic, there is a wide range of medicinal uses for onion in the studied material. According to folk belief, onions helped to create comfort and happiness in new homes and gave protection against evil powers. Medicinal uses included cures for ear diseases, warts, wounds and bee stings. Onion was also given to livestock during calving.

### Cultivation practices and uses of garlic, *Allium sativum* L.

There are just a few, rare records from the questionnaires of garlic being grown and used as a seasoning. Instead, garlic is mainly described as a medicinal plant with a wide range of uses for both humans and livestock, and also many uses founded in superstition and folklore. It is sometimes hard to distinguish between medicinal uses and folklore connected with this species.

#### Human medicine and folklore

Medicinal uses for garlic described in the material from the Nordic Museum and the Folklife Archives include colic, stomach diseases, boils, warts and lice (Table 5). One of the primary uses described in the Folklife Archives is to protect against evil powers such as ghosts, trolls, nixies and other beings from Scandinavian folklore. Many records describe garlic as important for the protection of new-borns before the christening [2] and the ritual known as churching for women who have just given birth. Garlic was often used for magical purposes in combination with, e.g. *vådastål*, a sharp object made of steel that has caused someone’s death or has broken during use (Table 6).

**Table 6:** Wild *Allium* species gathered as flavouring herbs and vegetables by the pre-industrial rural people in Sweden

Species	Swedish names
Chives, *A. schoenoprasum* L.	alvarlök, baislauk, gräslök, purlök, smörlök
Field garlic, *A. oleraceum* L.	backlök, baisklök, hundlök, kråklök
Ramson *A. ursinum* L.	rams, ramsk, ramslök, Brittas lök
Sand leek, *A. scorodoprasum* L.	kajp, kajpe, kep, skogslök, hundalök, gräslök, rokkenboll

#### Ethnoveterinary uses

Just as in human medicine, garlic was used as medicine for a wide range of diseases in cattle, horses, sheep, pigs and chickens. It was especially frequently used in the calving of cattle, and to protect sheep against foxes. It was just as commonly used for protection of livestock against evil powers as for medicinal purposes. Most records are from the south of Sweden; in the one record from the northern region of Medelpad, a respondent born in the 1860s describes how garlic was planted in the corners of the fields to protect the crop from evil powers [95]. Other records tell of old practices of planting garlic in secret places or stealing it from another man’s field [96]. Other records from Blekinge state that garlic shall be planted under a pear tree to protect against ‘fear’ [97]. Garlic was often given to livestock when they were put out to pasture as protection against both diseases and evil powers. Sometimes it was also buried beneath the floor in the stable as a cure for appetite loss. These hard-to-define practices are labelled as “other uses” in Table 5, together with protection against wild animals and a rare record of the use of garlic on the violin to strengthen the strings and put a spell on the musician [98].

The transition of garlic from being described as the one of the most common medicinal plants among the peasantry in the eighteenth century [99] to being less in cultivation due to its inclusion in pharmacy stock [96] is confirmed by the fact that in POM’s inventory there were only tree findings of garlics were made [18]. One of the accessions collected, described as *A. sativum* ‘Gusum’ (Fig. 3), was kept in the family as a memory of an old relative whose father bought the garlic from a pharmacy in a city nearby as a cure for his son’s stomach problems [18].

### Cultivation practices and uses of chives and other edible *Alliums*

In the historical literature studied and presented in Table 1, it is clear that the authors consistently describe chives, *A. schoenoprasum* L., as a vegetatively propagated crop. Chives are sometimes described as one of the most popular seasonings used by the Swedish peasantry [27, 96, 100], and the questionnaires confirm a range of cultivation practices and uses, even though it is not as commonly described as species like caraway, parsley and onions. One explanation for this might be that chives were a seasoning mainly used during the growing season as a premiere directly from the garden and not usually dried, conserved or traded (Fig. 2). The questionnaires do not give any significant information regarding the process by which chives changed from being considered as a vegetatively propagated crop in garden literature to a crop that is nowadays mainly propagated by seed. In records from Asarum parish in the province of Blekinge, a woman born in 1860 documents a statement from her father (born in 1827) about chives and another taxa called ‘roof onion’ (*taklök*), probably referring to *Sempervivum tectorum* L., although they are both referred to as onions [101]. Her father says that people in the area considered these plants as particularly tied to the caregiving role of women, and that women’s competence as housewives was judged by the lushness of these crops in the garden and on the roof. From the province of Västmanland, there is a description of chives being an important and popular vegetatively propagated crop in the early 1800s, but less popular by the end of the century [96]. The author explains that stands of chives that lost vitality had to be renewed, and that neighbouring housewives would cooperate in sharing the plants to avoid the shame of losing the crop.

#### Fish dishes and sauces

Chives are frequently used as a seasoning in recipes for fish dishes as garnish and in a variety of sauces, but especially as an important herb in staple dishes of potatoes, herring and pork. An account from Halland describes in detail the preparation of a meal commonly eaten in farmer’s homes the 1890s. Potatoes were cooked and then peeled as they were eaten. The potato was cut into slices and skewered on a knife, then dipped into a shared cup in the middle of the table containing creamed milk with salt and diced chives. In the other hand, a piece of pork or salted herring was held between thumb and forefinger and eaten in between bites of potato, before the fingers were licked clean [102].

Chives were used to flavour sauces of milk and flour that were served with simple dishes of pork, herring and potatoes in all parts of Sweden. Sometimes the questionnaire respondents do not distinguish between chives and other onions, which makes it possible to wrongly interpret some of the records concerning chives as onion, but it is still possible to see from the study that both onions and chives have had a similar use in these kinds of simple dishes.

#### ‘Butter onion’

The use of chives as a flavouring for butter is described in several records from southern Sweden [e.g. 103]. In summertime, fresh chives from the kitchen garden were mixed into butter to be used as a spread, but they were also used raw on the bread or to flavour grease from pork. The popular name ‘butter onion’ (*smörlök*) is sometimes given as an old folk name for chives. However, literature shows that this name has also been used for other taxa. Swedish author Fredrika Bremer [104] describes the onions eaten in church in Dalecarlia as ‘butter onion’ when travelling in the region in 1845, and earlier, in the firs printed list of Finland’s flora, *Catalogus plantarum* 1673, the common name was given to the taxon *Cepa candida* by Elias Tillandz [105], later defined as *Allium cepa* var. *flavescens* auct. by Lyttkens [91]*.* In Sollerön in Dalecarlia, onions grown like shallots, but called *vårlök* (‘spring onion’), were used to flavour old butter [62]. Not only might several taxa or culta of *Allium* have been eaten in church in different seasons and regions, but both chives and onions seem to have been used as ‘butter onions’.

#### Leek and other *Alliums*

Records of the use and cultivation of leek (*Allium ampeloprasum* Porrum-Group) are rare in the questionnaires. Its main documented use is in soups in southern Sweden, and it is often mentioned as an ingredient in meat dishes in records from the northern regions. From Dalsland, there is a record stating that leek was popular among the urban people while elderly rural people still used red and white onions as medicine against worms [106]. An account from Scania describes leek as being cultivated in gardens for use in soups. It was preserved by being chopped and mixed with salt, squeezed out and packed in a jar with salt between the layers [106].

There are rare occurrences in the questionnaires of *Alliums* being described using popular common names that are impossible to identify. Welsh onion, *A. fistulosum* L., is mentioned once in Scania together with another taxa that the respondent calls garlic, but does not recognise as what is commonly known as garlic [107]. From Blekinge, accounts of the cultivation of field garlic, *Allium oleracea* L., are given using the old name *hundlök* (‘dog onion’) [108]. This name is also used for chives [2]. The respondent proposes that this onion is still sometimes to be found in the wild, but has lost the qualities it used to have when it was cultivated and used in medicine. In Östergötland, *trollök* (‘troll onion’) is said to grow in the wild on the spot where an old troll (a mythological being in Scandinavian folklore) once lived and cultivated this kind of onion [103]. This taxon might be interpreted as referring to sandleek, *Allium scorodoprasum* L., which, as well as ramsons, *Alllium ursinum* L., is absent from the questionnaire responses.

## Discussion

The onion that, for instance, Carl Linnaeus called *Johanneslök* has had an unclear taxonomic affiliation, and the analysis of the results shown in Table 1 suggests that the term *Johanneslök* historically has referred to a special cultivation system where onion sets are planted for an early harvest, rather than to a specific taxon or species. German sources suggest that the similar German term *Johannis-Zwiebeln* also refers to a special cultivation system where onion sets are planted in the autumn to be harvested before Midsummer, or planted at midsummer to be harvested early the next spring. The absence of Welsh onion from both the questionnaires and POM’s inventory may suggest that cultons of this species were less commonly cultivated in the nineteenth century, or that the descriptions in the literature did not reflect actual cultivation practices of the time.

The description of a certain kind of shallot, especially suitable for Nordic conditions, first occurs in the literature in the 1850s (Table 1). The first use of the name ‘potato onion’ is found in the Swedish Garden Association catalogues from the 1830s when they were introduced from Europe, and referred to as both Russian and Egyptian [22]. Records from the questionnaires are coherent with other sources stating that the multiplying onions had gained great popularity among the peasantry in certain Swedish regions in the nineteenth century. The name *Leksandslök*, first appearing in 1859, gives reason to believe that there was locally adapted or selected plant material in cultivation before a more systematic introduction of cultivars occurred. The spread of the name ‘potato onion’ could not have preceded the popularity of the potato in the nineteenth century, and in the questionnaires is solely recorded from Scania.

In traditional food culture and folk botany, many species are classified and named by their uses, without consideration of taxonomic issues. Swedish folk names, such as *Pehrsmässolök* (‘Feast of Saint Peter’s onion’), *vårlök* (‘spring onion’), *syltlök* ‘pickled onion’, *smörlök* (‘butter onion’) and *Johannislök* (‘Saint John’s onion’) can be understood similarly and represent a rational way of categorising knowledge at a time when the principle of cultivars was not consistently understood or used for all crops. Differing naming principles make it difficult to interpret historical records, but at the same time, give valuable information about the various uses and qualities associated with a particular species. Morphologically descriptive trivial names of both species and early cultivars provide insights into variation in the plant material and the qualities associated with it; names referring to uses or practices shed light on cultivation systems and food traditions. Taxonomic confusion surrounding the ‘Saint John’s onion’ searched for by Linnaeus and Miller’s “true scallion”, and the notion that there was an old species that had been lost, point to a continuity in the mystique surrounding edible *Alliums* originating from older times and/or cultures. The later introduction of the term ‘potato onion’ and the notion that old plant material preserved by the peasantry held lost qualities are examples of how shows that the documentation and name use connected to plant material adds to its conservation value, and aids understanding of the material’s worth.

The species of onion discussed in this paper are examples of an important cultural heritage, which is of interest to, for instance, ethnobiologists and garden historians. It is also important to preserve them for the future. The old varieties and landraces are well adapted and well suited to local production systems, and they offer increased resilience for crop production. They are therefore potential resources for crop improvement, especially for the development of varieties that are tolerant to biotic and abiotic stresses and for incorporating farmer-preferred traits. It is our firm belief that old cultivated plants are an important resource in the search for the food of the future.

## Conclusion

Old cultivars and landraces are not only of historical interest; they may also be of interest for future food supply. Genetic diversity of vegetables and garden crops represents a critical resource to achieve and maintain global food security. The disparate findings regarding cultivation practices and use found across a variety of sources—historic garden literature, questionnaires conducted in the early twentieth century, and modern-day inventories—indicate that some plant knowledge might be neglected if research methods are too narrow. Examples from this study include the fact that the use of onions as an important ingredient in charcuteries is reflected neither in POM’s inventory nor in the historical garden literature, while the practice of autumn planting only appears in the literature and not in the questionnaires. In POM’s inventory, the practice of linking childhood memories and stories told by older relatives to heirloom onions and other plants, and the fact that these were sometimes described as superior to modern varieties, exemplify how different questionnaires and inventory methods might reflect the values of the period in which they are performed. The overlapping findings show that different ways of reproducing old knowledge are in themselves an important element of the cultural heritage of cultivated plants that need to be further investigated, and also understood as part of our green heritage.

## Data Availability

All data generated or analysed during this study are included in this published article.
